# Alteration in the Gut Microbiota Provokes Susceptibility to Tuberculosis

**DOI:** 10.3389/fimmu.2016.00529

**Published:** 2016-11-28

**Authors:** Nargis Khan, Aurobind Vidyarthi, Sajid Nadeem, Shikha Negi, Girish Nair, Javed N. Agrewala

**Affiliations:** ^1^Immunology Division, CSIR-Institute of Microbial Technology, Chandigarh, India

**Keywords:** antibiotics, gut microbiota, tuberculosis, *Mycobacterium tuberculosis*, fecal transplant

## Abstract

The microbiota that resides in the gastrointestinal tract provides essential health benefits to the host. In particular, they regulate immune homeostasis. Recently, several evidences indicate that alteration in the gut microbial community can cause infectious and non-infectious diseases. Tuberculosis (TB) is the most devastating disease, inflicting mortality and morbidity. It remains unexplored, whether changes in the gut microbiota can provoke or prevent TB. In the current study, we have demonstrated the antibiotics driven changes in the gut microbial composition and their impact on the survival of *Mycobacterium tuberculosis* (*Mtb*) in the lungs, liver, and spleen of infected mice, compared to those with intact microbiota. Interestingly, dysbiosis of microbes showed significant increase in the bacterial burden in lungs and dissemination of *Mtb* to spleen and liver. Furthermore, elevation in the number of Tregs and decline in the pool of IFN-γ- and TNF-α-releasing CD4 T cells was noticed. Interestingly, fecal transplantation in the gut microbiota disrupted animals exhibited improved Th1 immunity and lesser Tregs population. Importantly, these animals displayed reduced severity to *Mtb* infection. This study for the first time demonstrated the novel role of gut microbes in the susceptibility to TB and its prevention by microbial implants. In future, microbial therapies may help in treating patients suffering from TB.

## Introduction

Approximately one-third of the world population is infected with *Mycobacterium tuberculosis* (*Mtb*), but only 5–10% contract active tuberculosis (TB), whereas the remaining 90–95% develop effective immunity (1). An intriguing possibility is that there exists an intricate balance between host and pathogen; where the host develops remarkably powerful immunity, which does not allow the pathogen to replicate and inflict disease. However, any disturbance in this finely tuned balance may lead to the development of TB.

The gut microflora is an immense health asset for human beings (2). The mammalian gut harbors trillions of commensals. These microbes influence not only local but also systemic immunity. Recently, various reports signify that the gut microbes can modulate, tune, and tame the host immune response (3). Importantly, an ever-growing number of disorders have been linked with resident microbiota and gastrointestinal diseases, such as intestinal bowel disease (IBD) (4). More importantly, imbalance in the gut microbiome has been shown to be associated with extra-intestinal ailments such as cancer, cardiovascular diseases, obesity, and non-alcoholic fatty liver disease (5–8). Consequently, it advocates the significance of the microbial composition that can influence our health. The microbiota provides a fine equilibrium to the host by regulating immune homeostasis (9).

Antibiotics are often used in the clinics to treat bacterial infections, but they are also major factor in disturbing the gut microbial composition (10). Antibiotics driven changes in gut microbiota provoke host susceptibility to enteric infection. However, impact of antibiotics induced changes in gut microbiota on the TB progression has not yet been studied. Therefore, we designed our study taking into consideration two models pre- and post-antibiotics treatment models of experimental TB. In pre-antibiotics model, animals were treated with broad spectrum antibiotics prior to *Mtb* infection, mimicking a condition wherein the individuals undergo treatment with various antibiotics before being exposed to *Mtb* and may have some impact on the progression of TB. In post-antibiotics model, animals were treated with antibiotics after *Mtb* infection. Post-antibiotics treatment study was designed to consider those individuals who are exposed to *Mtb* and take broad spectrum antibiotics for some other infections. Interestingly, we observed that animals with disruption in gut microbiota after both pre- and post-antibiotics treatment showed higher susceptibility toward *Mtb* and promoted its dissemination. Intriguingly, fecal transplant (FT) from normal mice reconstituted the gut microbiota of antibiotics treated animals, which subsequently decreased the *Mtb* load in the lungs and prevented the dissemination of the disease. In essence, this finding signifies that the alteration in the gut microbiota may facilitate the development of TB.

## Materials and Methods

### Animals

C57BL/6 mice, 6–8 weeks were procured from the CSIR-Institute of Microbial Technology (IMTECH), Chandigarh, India.

### Ethics Statement

All the experiments were approved by the Institutional Animal Ethics Committee of the IMTECH and performed according to the National Regulatory Guideline issued by Committee for the Purpose of Supervision of Experiments on Animals (No. 55/1999/CPCSEA), Ministry of Environment and Forest, Government of India.

### Cultivable Microbes

Fecal samples (200–300 mg) were collected aseptically in 1 ml of 1× PBS. Samples were homogenized and supernatant were collected after centrifuging samples at 2000 rpm for 2 min to pellet down debris. Later, serial dilutions were made and supernatants (100 μl) were plated on different media to culture both aerobic and anaerobic microbes. To cultivate anaerobic microbes, plates were kept in vacuum tight container in the presence of anaerobic gas pack (Himedia, Mumbai, India) for overnight.

### Bacterial Diversity

To assess the bacterial diversity, supernatant from fecal samples was plated on different media to culture both aerobic and anaerobic microbes for overnight, as described above. The identification of colony morphotypes was carried out using four parameters: colony size, form, color, and texture. A phenotypic variant was considered when it differed in at least one of the referred morphological parameters. Diversity was calculated by counting the total number of different colonies of bacteria grown on different media plates. Total different colonies from the feces of healthy animals were considered as a reference number. Decrease in the bacterial diversity was calculated as (reference number − total different colonies calculated from infected or pre-antibiotics or post-antibiotics) × 100/reference number.

### Pre-Antibiotics-Treated *Mtb* Model (Pre-Antibiotics)

Mice were pre-treated with vancomycin (100 mg/l), polymixin B (60 mg/l), carbenicillin (50 mg/l), trimethoprim (20 mg/l), and amphotericin B (50 mg/l) *ad libitum* in drinking water for 21 days. The water containing antibiotics were replaced on every 3 days. Control mice were fed antibiotics free water. After 21 days, mice were challenged with *Mtb* (H37Rv) with deposition of 100 CFU in the lungs. The animals were administered antibiotics in the water for additional 21 days. Later, the mice were sacrificed, and tissues were harvested aseptically for immunological, microbiological, and histopathological studies. Pictorial representation of methodology is embedded in Supplementary Material.

### Post-Antibiotics-Treated *Mtb* Model (Post-Antibiotics)

Mice were challenged with *Mtb* with the deposition of 100 CFU in the lungs. After 21 days, mice were treated with antibiotics for subsequent 21 days with replacement of water every 3 days. Later, mice were sacrificed, and tissues were harvested aseptically for immunological, microbiological, and histopathological studies. Pictorial representation of methodology is embedded in Supplementary Material.

### Reconstitution of Gut Microbiota

Antibiotics-treated mice underwent FT to reconstitute the gut composition. Fecal samples (200–300 mg) from five healthy mice were collected aseptically in five separate tubes in 1 ml of 1× PBS. Samples were homogenized and supernatant was collected after centrifuging samples at 2000 rpm for 2 min to pellet down debris. Supernatant slurry was collected and pooled together, and 100 μl was gavaged into mice within 15–20 min of excretion. Five doses with a gap of 3 days interval were orally administered to mice prior to 15 days of sacrificing animals. Pictorial representation of methodology is embedded in Supplementary Material as Methodology 2.

### Expression of IFN-γ, TNF-α, and Foxp3 by Flowcytometry

Spleen was harvested, and single-cell suspension was prepared. Briefly, lymphocytes from spleen were prepared by lysing RBCs with ACK lysis buffer (NH_4_Cl 0.15M, KHCO_3_ 10 mM, EDTA 88 mM), washed 3× with PBS, and resuspended in RPMI-1640-FBS-10%. Viability of the cells was assessed by trypan blue dye-exclusion method. The experiments were performed to detect intracellular cytokines and Foxp3 expression on T cells. To detect cytokines, splenocytes were restimulated *in vitro* with purified protein derivative (PPD). Splenocytes stimulated with PPD (20 μg/ml) were cultured for 48 h at 37°/CO_2_ (5%). During last 4 h, cells were incubated with phorbol 12-myristate 13-acetate (PMA; 20 ng/ml) and ionomycin (1 μM) plus brefeldin A (5 μg/ml) for 2 h. The cells were stained with anti-CD4 Abs. After surface staining, cells were washed and resuspended in permeabilization-fixation solution (BD Cytofix/Cytoperm kit; BD Pharmingen, San Diego, CA, USA), and intracellular cytokine staining was performed with fluorescence-labeled Abs to TNF-α, IFN-γ, according to manufacturer’s protocol. FoxP3 staining was performed (Foxp3/Transcription Factor Staining Buffer Set, Ebioscience) according to manufacturer’s instructions. Data were collected using FACS Aria and analyzed using the BD DIVA software.

### Statistical Analysis

Statistical analysis was done using unpaired Student’s *t*-test and one-way ANOVA for group analysis using the Graph pad prism software 6.

## Results

### Antibiotics Treatment Modulates the Gut Microbiota Composition

**Figure 1:**
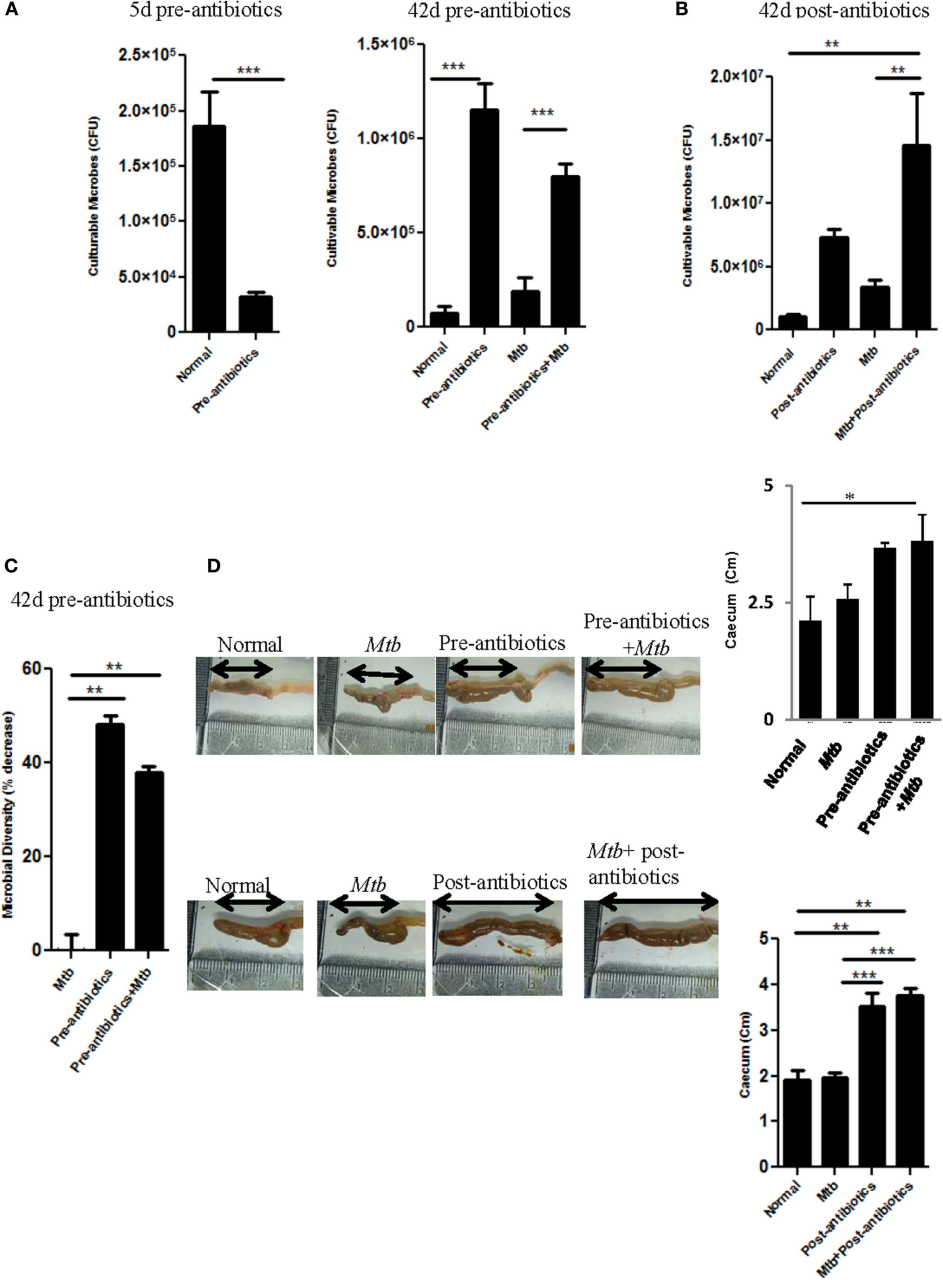
**Antibiotics altered the gut microbial composition**. **(A–C)** Mice were pre-treated with broad spectrum of antibiotics for 42 days. In between, on day 21, animals were challenged with *Mtb*. **(A)** Cultivable microbes on 5 and 42 days; **(C)** microbial diversity on 42 days were enumerated in fecal samples of treated mice. **(B)** In post-antibiotics model after 21 days of *Mtb* challenge, mice were treated with antibiotics daily for subsequent 21 days. Later, cultivable microbes were enumerated in the fecal samples. **(D)** Increment in the size of cecum in both pre-antibiotics and post-antibiotics animals was measured; bar graph represents the length (centimeters) of cecum. Data shown as mean ± SEM are representative of three independent experiments (*n* = 4–5 animals/group) (*p < 0.05, **p < 0.01, and ***p < 0.001).

Recent studies have shed new light on an impact of antibiotics on the gut microbes, which eventually may affect the severity of disease. Hence, we were curious to explore the influence of altered gut microbiota on the progression of TB. To test this hypothesis, mice were fed with broad spectrum antibiotics, prior to *Mtb* infection (pre-antibiotics) (for detailed methodology, please see Supplementary Material). Broad spectrum antibiotics were selected for the study. These antibiotics exhibited no impact on *Mtb* recovery in a Middlebrook 7H11 agar formulation (11). Animals treated with antibiotics for initial 5 days showed significant (*p* < 0.001) decrease in the CFU of the gut microbiota (Figure 1A). In contrast, when the antibiotics treatment was prolonged for 42 days, significant (*p* < 0.001) elevation in the number of microbes was observed (Figure 1A). Similar results (*p* < 0.01) were observed in the case of mice prior challenged with *Mtb* and then treated with antibiotics (post-antibiotics) (Figure 1B). Results suggest that initially antibiotics sensitive bacteria were eliminated, which resulted in the decline of CFUs (Figure 1A). However, later antibiotics resistant microbes predominated by over proliferation. However, we observed significant (*p* < 0.01) decrease in the diversity of the microbial taxa (Figure 1C), since the majority of the colonies were of sister clones, as identified through their morphology. Furthermore, we noticed enlargement in the size of cecum (Figure 1D). This change in the intestine was due to the decreased ability of antibiotics-treated mice to digest the food, as it was apparent by the presence of indigested food in the intestine. Similar results were observed in post-antibiotics-treated *Mtb* animals (Figure 1D).

### Animal Treated with Antibiotics Showed Higher *Mtb* Burden in the Lungs and Its Dissemination

**Figure 2:**
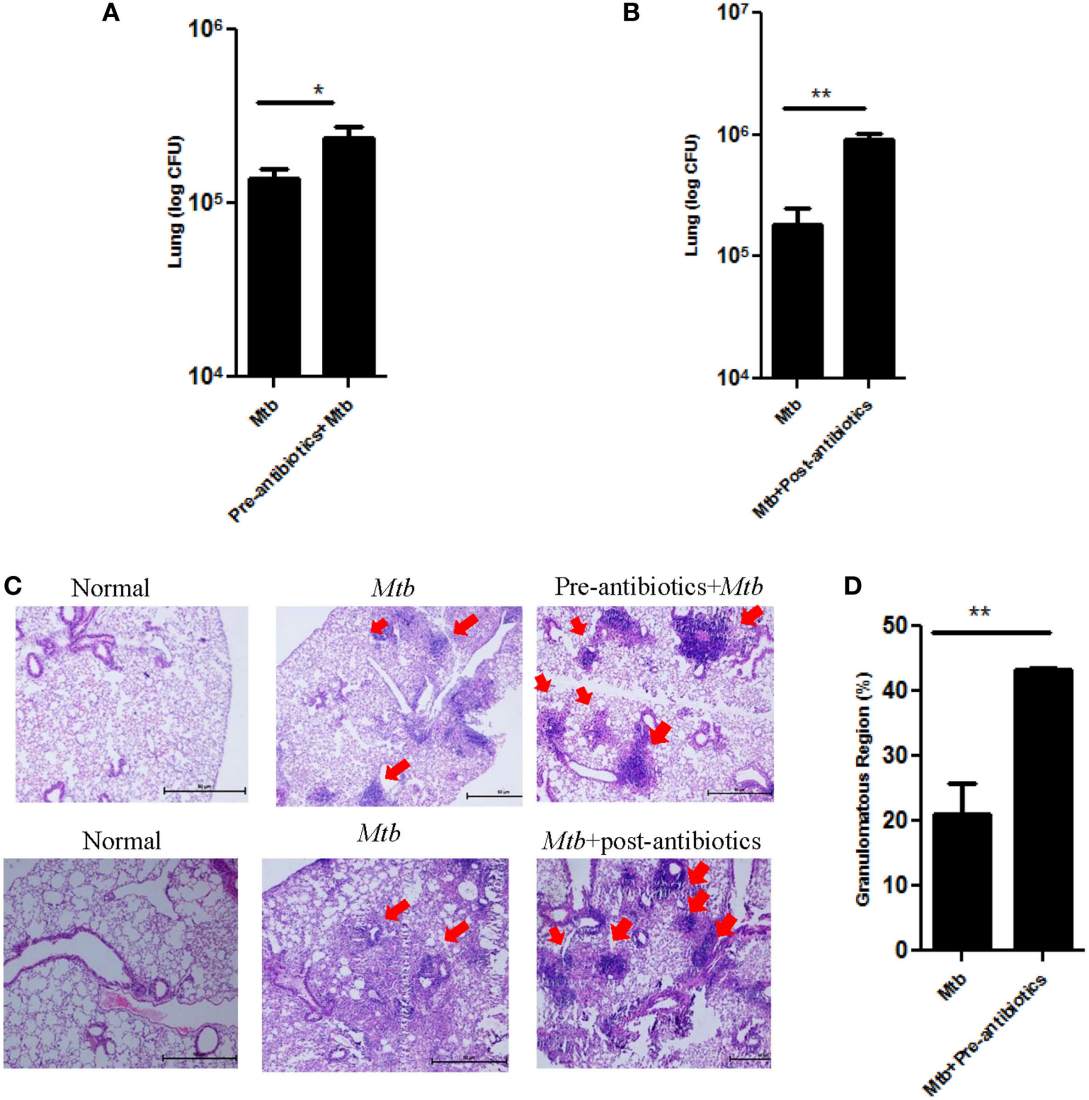
**Disturbance in gut microbiota by antibiotics increased the survival of *Mtb* in the lungs and its dissemination to other organs**. Mice treated with antibiotics prior and post exposure to *Mtb* infection. Later, animals were sacrificed, and lungs were isolated. Bacterial burden in **(A)** pre-antibiotics and **(B)** post-antibiotics-treated group was estimated by plating serial dilutions of lung homogenate on 7H11 agar plates. Colonies were enumerated on 21 days of plating. Bar graph depicts the bacterial burden in lungs. Data shown as mean ± SEM are representative of three independent experiments (*n* = 4–5 mice/group). **(C)** Histopathology sections of lungs of pre-antibiotics and post-antibiotics group were H&E stained and imaged at a magnification 40×. **(D)** Bar graphs depict the percentage of tuberculous region of pre-antibiotics *Mtb* group. Data are representative of three independent experiments (*n* = 4–5 animals/group) (*p < 0.05 and **p < 0.01).

The aim of the study was to assess the consequence of antibiotics driven alteration in the gut microbiota on *Mtb* survival in *Mtb* challenged mice. Interestingly, we observed that disruption of microbiota in pre-antibiotics (*p* < 0.05) and post-antibiotics (*p* < 0.01) treatment, significantly enhanced the growth of *Mtb* in the lungs of the infected animals (Figures 2A,B). This information was further corroborated with histopathological analysis of the lungs (Figure 2C). *Mtb*-infected mice on pre-antibiotics treatment showed larger and greater number of granulomas in their lungs, compared to control (*Mtb* infected, but not treated with antibiotics) (Figure 2C, upper panel). In addition, we observed significant increase in the granulomatous or tuberculous region (*p* < 0.01) in lungs of pre-antibiotics *Mtb*-infected animals (Figure 2D). Similar trend was observed in the post-antibiotics-treated animals as shown in histopathological images (Figure 2C, lower panel).

**Figure 3:**
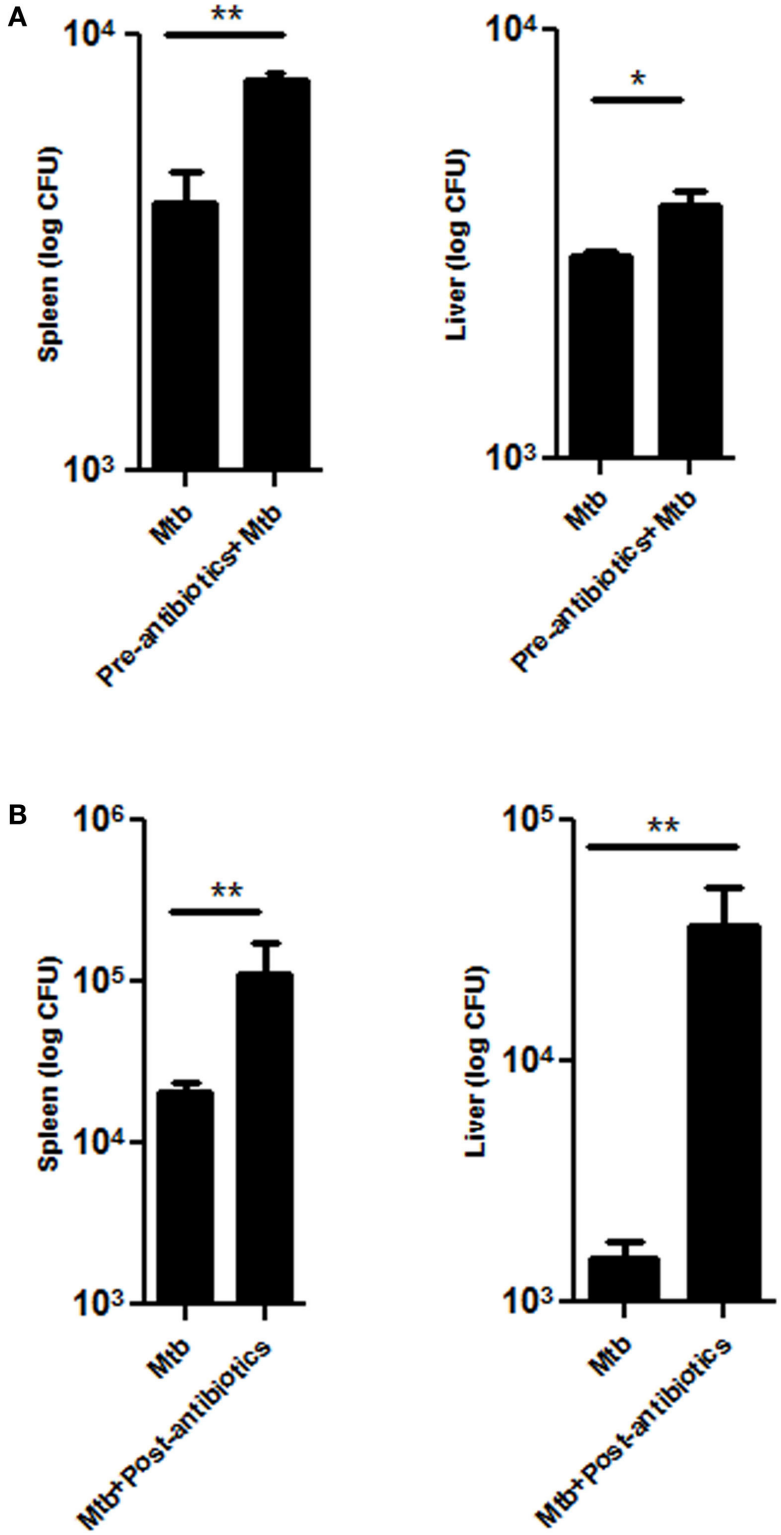
**Disrupted gut microbiota promotes the dissemination of *Mtb***. Mice were treated with **(A)** pre-antibiotics and **(B)** post-antibiotics to *Mtb* infection. After 42 days, mice were sacrificed, and *Mtb* load was estimated in the spleen and liver by enumerating CFUs. Bar graph depicts the *Mtb* load. Data shown as mean ± SEM are representative of three independent experiments (*n* = 4–5 animals/group) (*p < 0.05 and **p < 0.01).

Dissemination of *Mtb* from lungs to other parts of the body is a probable factor responsible for extra-pulmonary infection. Interestingly, significant increase in the bacterial load of *Mtb* was observed in the spleen (*p* < 0.01) and liver (*p* < 0.05) of pre-antibiotics *Mtb*-infected model (Figure 3A). Similarly, post-antibiotics infected mice showed greater *Mtb* burden in the spleen (*p* < 0.01) and liver (*p* < 0.01), as compared to controls (Figure 3B). These data suggest that gut the microbiota play a crucial role in restricting the proliferation and dissemination of *Mtb*.

### Fecal Transplantation in Antibiotics-Treated Mice Reconstitutes the Gut Microbiota

**Figure 4:**
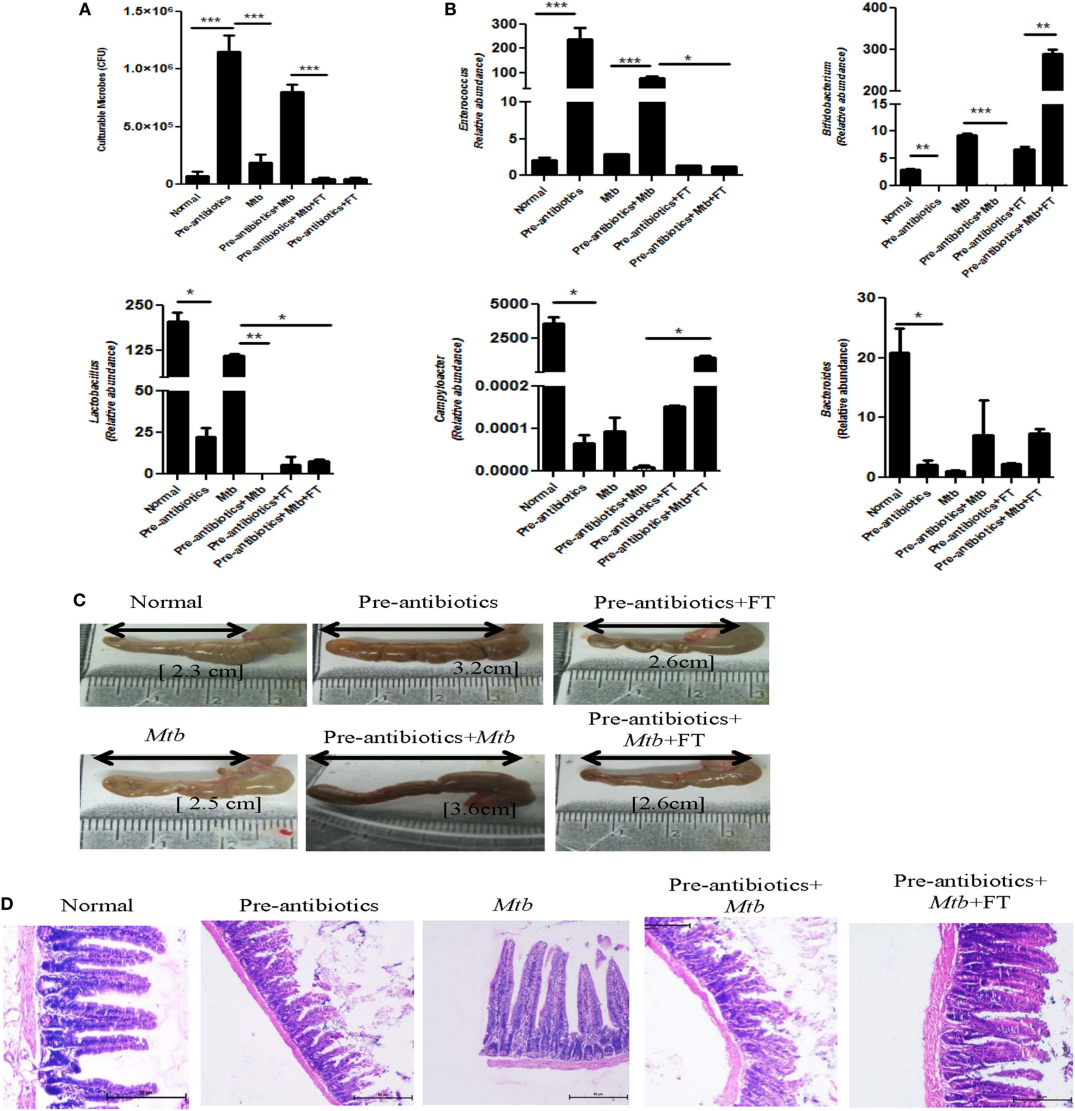
**Fecal transplantation reconstitutes the gut microbiota**. **(A)** Pre-antibiotics mice were administered five doses of FT, 15 days prior to sacrificing animals. Later, **(A)** enumeration of cultivable microbes was assessed in the fecal samples. **(B)** Alteration in the number of microbes, such as *Enterococcus, Bifidobacterium, Lactobacillus, Campylobacter*, and *Bacteroides*, was studied in the fecal samples by RT-qPCR. **(C)** Increment in the size of cecum was measured. **(D)** Histopathology sections of ileum were H&E stained and photomicrographs are shown at 100× magnification. Data shown as mean ± SEM are representative of two independent experiments (*n* = 4–5 animals/group) (*p < 0.05, **p < 0.01, and ***p < 0.001).

Before sacrificing animals, both pre-antibiotics and post-antibiotics-treated groups were fecal transplanted orally. Interestingly, we observed significant (*p* < 0.001) decline in the number of gut microbes in pre-antibiotics model after fecal transplantation and their number was comparable with normal mice (Figure 4A). Furthermore, there was significant (*p* < 0.001) but partial restoration in the microbial diversity, as noticed through morphology of microbes in FT group (Figure S1 in Supplementary Material). The variation was not resilient, i.e., did not return to its original percentage. It suggests that lowering of microbial diversity by antibiotics treatment could not be fully restored to its original frequency after FT for 15 days. It is reported that antibiotics treatment decreases the number of beneficial microbes (12). Therefore, we have confirmed the alteration in gut microbiota due to antibiotics treatment, by identifying microbes in the fecal samples by RT-qPCR. We observed significant increase in the number of *Enterococcus* (*p* < 0.05) but decline in the level of *Bifidobacterium* (*p* < 0.01), *Lactobacillus* (*p* < 0.05), *Campylobacter* (*p* < 0.05), and *Bacteroides* (*p* < 0.05) (Figure 4B). These results further support the partial reconstitution of gut microbial composition through FT. This information was further corroborated by PCR (Figure S2 in Supplementary Material). As compared to control animals (2.3 cm), antibiotics-treated group showed increase in the size of cecum (3.2 cm). Interestingly, FT restored the size of cecum to near normal (2.6) (Figure 4C). Furthermore, as compared to controls, the histopathological studies conducted on the ileum of pre-antibiotics-treated animals exhibited distorted structure of microvilli. The microvilli structure of ileum was reinstated to normal in the FT animals (Figure 4D). Similar results were observed in post-antibiotics model (Figure S3 in Supplementary Material). It was interesting observation that FT recovers antibiotics associated abnormities.

### Fecal Transplantation Restricts the Growth as well as Dissemination of *Mtb*

**Figure 5:**
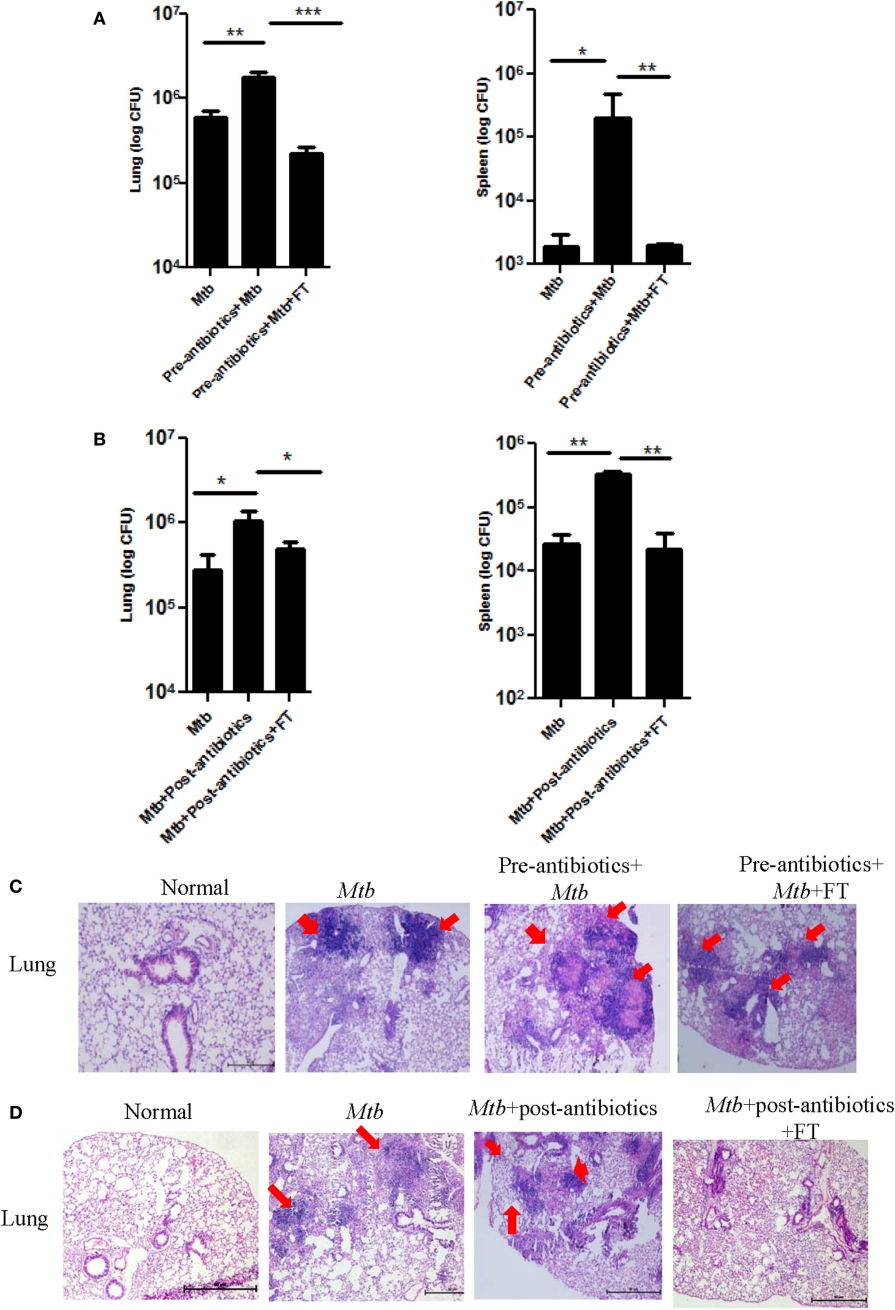
**Restoration of gut microbiota restrains the growth of *Mtb* in the lungs and prevents its dissemination**. Mice with **(A,C)** pre-antibiotics and **(B,D)** post-antibiotics treatment were provided five doses of FT, 15 days prior to sacrificing. Later, *Mtb* load was estimated in lungs and spleen. Bar graph depicts the *Mtb* load. Histopathology sections of lungs of **(C)** pre-antibiotics and **(D)** post-antibiotics-treated group were H&E stained and imaged at 40× magnification. Data shown as mean ± SEM are representative of two independent experiment (*n* = 5 animals/group) (*p < 0.05, **p < 0.01, and ***p < 0.001).

Since, we observed significant augmentation in the growth of *Mtb* in the lungs of pre-antibiotics (*p* < 0.05) and post-antibiotics (*p* < 0.01) groups (Figures 2 and 3), therefore we thought to study the influence of FT on pre-antibiotics and post-antibiotics animals infected with *Mtb*. Interestingly, FT significantly reduced the bacterial load in the lungs (*p* < 0.001) and spleen (*p* < 0.01) of both pre- and post-antibiotics mice exposed to *Mtb* (Figures 5A,B).

We also examined the progression of disease by studying the histopathological changes in the lungs. As compared to control group, larger size and number of granulomas in the lungs of pre- as well as post-antibiotics group were noted (Figures 5C,D). It was observed that antibiotics-treated mice after FT, exhibited granulomas with smaller size and number, lesser infiltration of lymphocytes, and consolidated lung architecture, compared to antibiotics group (Figures 5C,D). These results suggest that gut microbiome can contribute in controlling *Mtb* growth and its dissemination.

### Antibiotics Treatment Augments Tregs but Suppresses Th1 Cells

**Figure 6:**
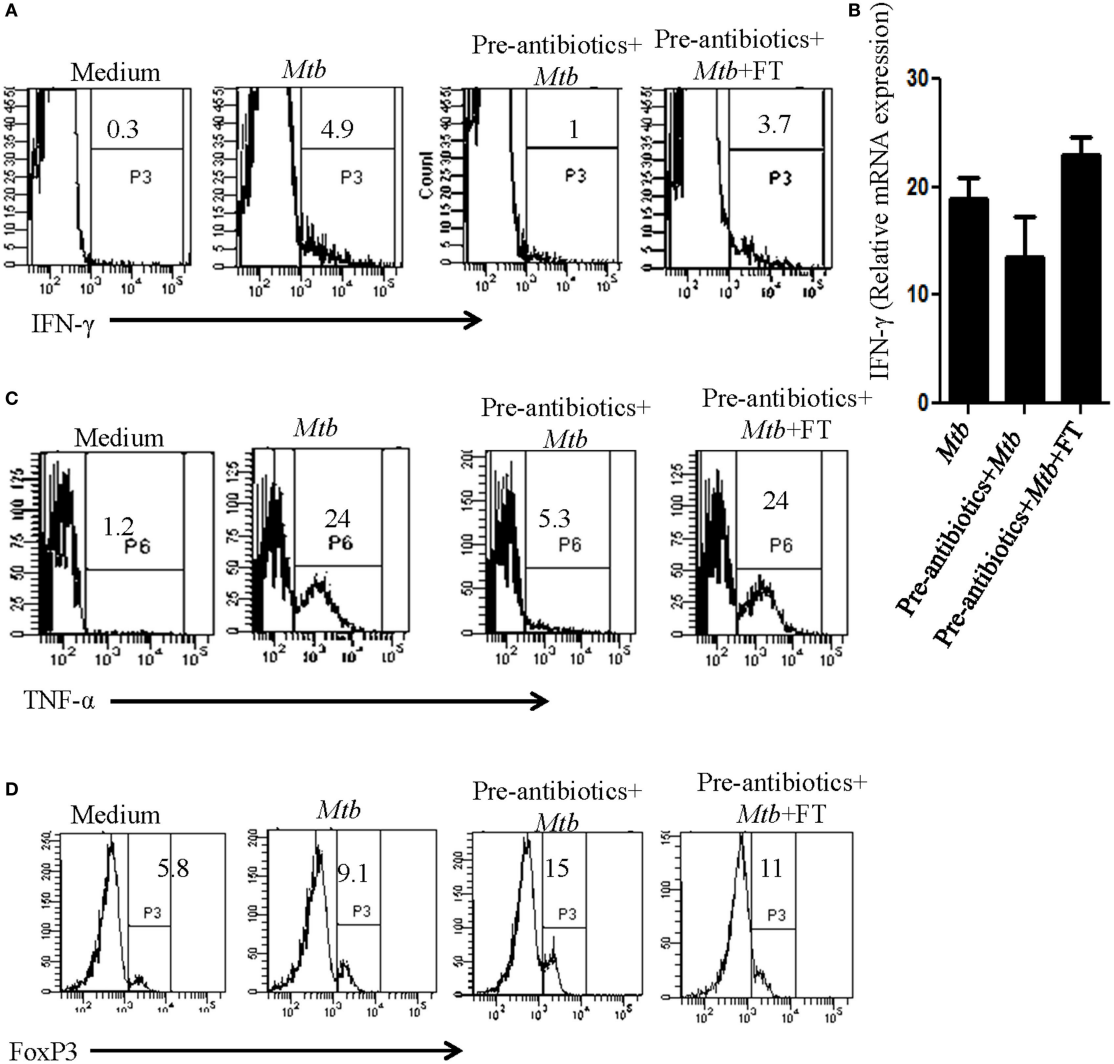
**Dysbiosis of gut microbiota imbalanced the frequency of Th1 and Tregs cells**. Mice with pre-antibiotics treatment were provided five doses of FT, 15 days prior to sacrificing. Later, intracellular expression of **(A)** IFN-γ, **(C)** TNF-α, and **(D)** FoxP3 was monitored in CD4-gated T cells by flowcytometry. **(B)** IFN-γ expression was detected at mRNA level by RT-qPCR. Data shown are representative of two independent experiments (*n* = 4–5 mice/group).

The role of Th1 cells is established in protection, whereas Tregs promote susceptibility to TB. Consequently, it was imperative to analyze the impact of antibiotics treatment on Tregs and Th1 cells and correlation in the modulation in their frequency with predisposition to TB. We observed substantial decline in the expression of IFN-γ and TNF-α upon treatment with antibiotics (Figures 6A,C). It was of interest to note the restoration of the production of IFN-γ and TNF-α in the mice with FT (Figures 2 and 3). Expression of IFN-γ in the spleen was further confirmed by RT-qPCR (Figure 6B). In contrast, antibiotics treatment augmented the frequency of Tregs, whereas FT downregulated their number as evidenced by the expression of Foxp3 (Figure 6D). This observation suggests that the antibiotics driven fluctuation in the gut microbiota can modulate the frequency of Tregs and Th1 cells, which may be responsible for proneness to TB.

## Discussion

Tuberculosis is one of the world’s leading killer diseases with approximately two million deaths and eight million new cases annually. It is public health and economic burden on the country (13). However, majority of *Mtb* exposed individuals remain asymptomatic but exhibit varying level of immunity against *Mtb* infection. Several host factors have been identified in both mice and humans that contribute to susceptibility toward *Mtb* infection. The *nramp1/SLC11A1* confers resistance in the murine model of TB, typhoid, and leishmaniasis (14). The role of diet also considerably contributes in prompting disease symptoms (15).

Recently, research related to gut microbiota has gain considerable impetus, following the observation of its correlation with many immune disorders (16, 17). Shifts in the composition of the microbiota, whether induced by dietary changes, antibiotics treatment, or invasive pathogens, can disturb the balance of gut microbes and dysregulate the function of local as well as systemic immune system (9, 18, 19). Perturbation in the gastrointestinal microbiota composition is also strongly associated with allergies and asthma (12, 20).

Current study revealed the role of gut microbiota in controlling the pathogenesis of TB. Key findings emerged from the study suggest that disruption of gut microbiota with antibiotics of *Mtb*-infected animals revealed: (i) significant alteration in the gut microbiota; (ii) higher *Mtb* burden in the lungs; (iii) dissemination of *Mtb* to spleen and liver; and (iv) fecal implants reconstituted the gut microbiota and recuperated TB by declining the *Mtb* burden.

We selected antibiotics that were effective against both Gram-positive and -negative bacteria. Furthermore, dose of antibiotics was carefully chosen that showed no effect on *Mtb* viability, even when administrated for 42 days. Importantly, the selected dose significantly induced dysbiosis in the gut. Importantly, alteration in gut microbiota promotes the survival of *Mtb* in the lungs. This finding emphasizes that composition and function of the gut community are important factors in conferring host resistance to invading pathogens. Our study revealed very interesting findings that antibiotics mediated disruption of gut microbiota not only increases the growth of *Mtb* in the lungs but also promotes its dissemination to other organs. Impact of antibiotics driven changes in gut microbiota on *Mtb* survival was further supported by fecal transplantation. Interestingly, fecal transplantation of antibiotics-treated mice restores the gut microbiota and decreases the *Mtb* burden in their lungs and prevents dissemination to spleen.

Microbiota plays an active role in the development and function of both pro- and anti-inflammatory T-cell pathways (21, 22). Frequency of the gut microbiota should be well tuned to mount host response against pathogens. Imbalance in the number of Tregs changes the microbial composition and *vice versa* (23). CD4 T cells are the major players in imparting immunity against *Mtb*. It is important to mention that the gut microbiota contributes substantially in the development of CD4 T cells, both within and outside the intestine (12, 24). We also observed that mice treated with antibiotics showed suppression of Th1 immunity but increase in the frequency of Tregs. Fascinatingly, fecal transplantation of antibiotics-treated mice restored the gut microbiota and reinvigorated immunity by augmenting the pool of IFN-γ and TNF-α releasing Th1 cells. In contrast, inhibition in the population of Tregs was noted. Currently, it is difficult to explain how this phenomenon is operating. However, this observation is quite interesting and may open a new line of investigation.

Antibiotics have been a cornerstone of innovation in the field of public health, but their negative effect on immune system and health cannot be ignored. Antibiotics driven compositional changes in the intestinal microbiota lead to severe dysregulation in the physiological and immunological intestinal homeostasis, creating serious and adverse consequences for the host (25, 26). To overcome such effect, new treatment strategies should be developed and designed to counteract the negative effect of antibiotics. One strategy could be to provide probiotics to supplement antibiotics induced deficits in the microbiota. Another, yet better approach could be to use immunomodulators to enhance the efficacy of immune system to combat infectious agents. With better understanding of the correlation between gut microorganisms and *Mtb*, one hope is that their manipulation/supplementation might prove to be a future targeted therapy for treating diseases. Advances in understanding how gut microbiota regulates the pathogenesis of TB, may pave a novel way toward new therapeutic intervention.

## Author Contributions

Concept or design of the work by JA and NK; experiments performed by NK, AV, SN, SkN, and GN; and analysis or interpretation of data for the work by NK and JA.

## Conflict of Interest Statement

The authors declare that the research was conducted in the absence of any commercial or financial relationships that could be construed as a potential conflict of interest. The reviewer AH and handling Editor declared their shared affiliation, and the handling Editor states that the process nevertheless met the standards of a fair and objective review.
