# Supplementary material for: Alteration in the Gut Microbiota Provokes Susceptibility to Tuberculosis
Source: Front Immunol. 2016 Nov 28;7:529. doi: 10.3389/fimmu.2016.00529 (PMC5124573; doi:10.3389/fimmu.2016.00529)
Supplement: Supplementary file 1 [file Presentation_1.PPTX]

## Slide 1
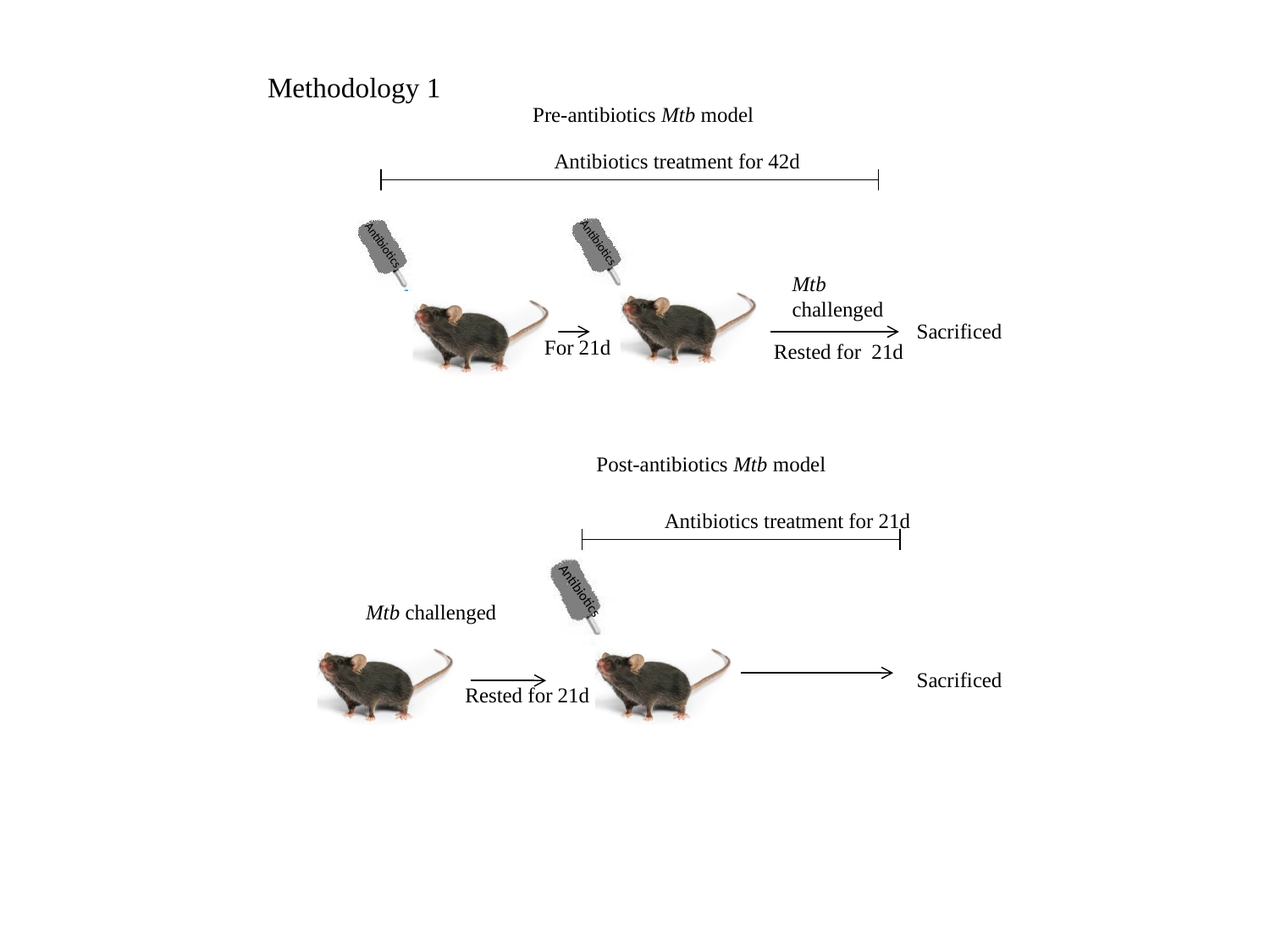

Methodology 1
Pre-antibiotics Mtb model
Antibiotics treatment for 42d
Antibiotics
Antibiotics
Mtb challenged
Sacrificed
For 21d
Rested for 21d
Post-antibiotics Mtb model
Antibiotics treatment for 21d
Antibiotics
Mtb challenged
Sacrificed
Rested for 21d

## Slide 2
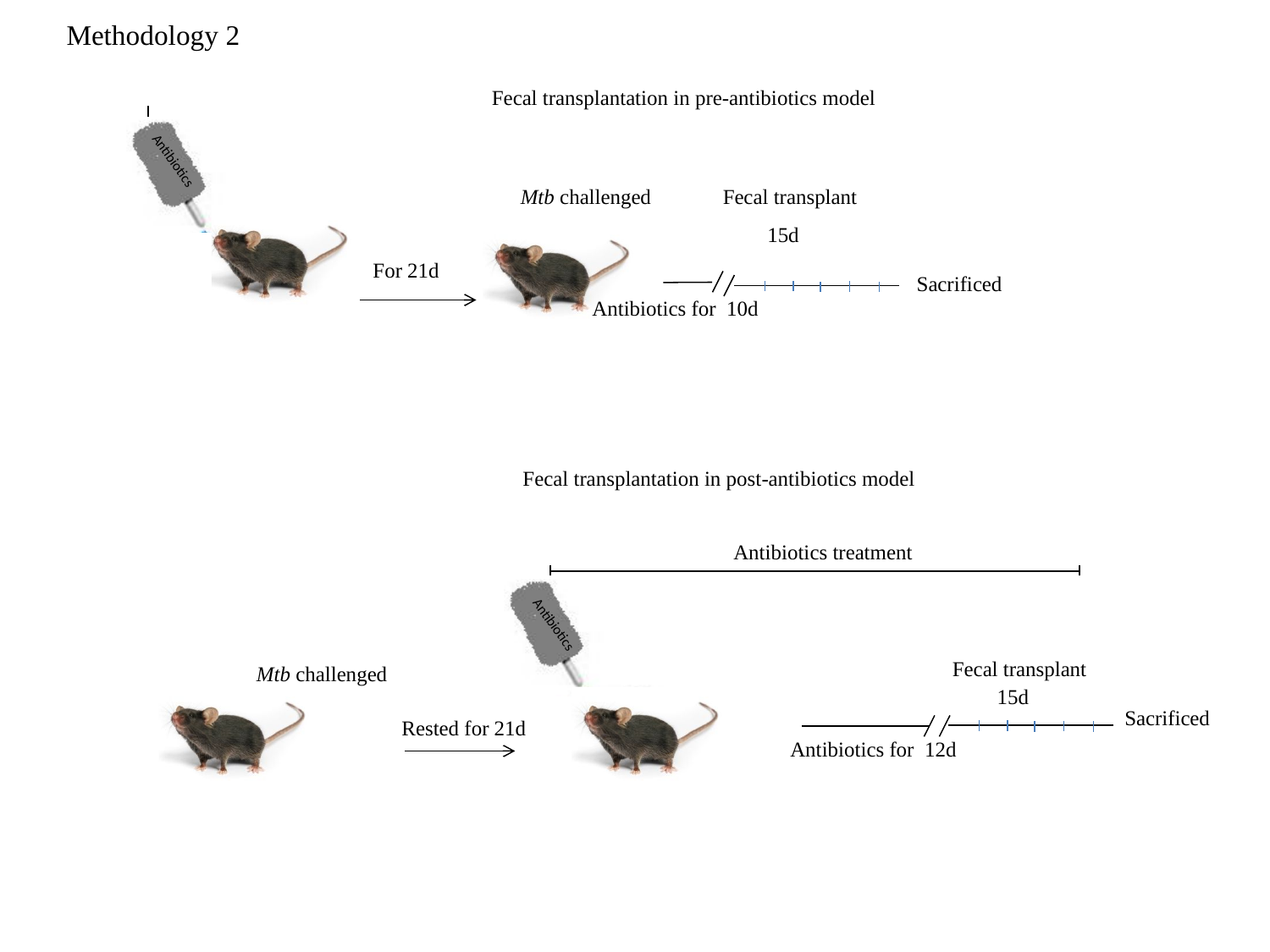

Methodology 2
Fecal transplantation in pre-antibiotics model
Antibiotics
Mtb challenged
Fecal transplant
15d
For 21d
Sacrificed
Antibiotics for 10d
Fecal transplantation in post-antibiotics model
 Antibiotics treatment
Antibiotics
Fecal transplant
Mtb challenged
15d
Sacrificed
Rested for 21d
Antibiotics for 12d

## Slide 3
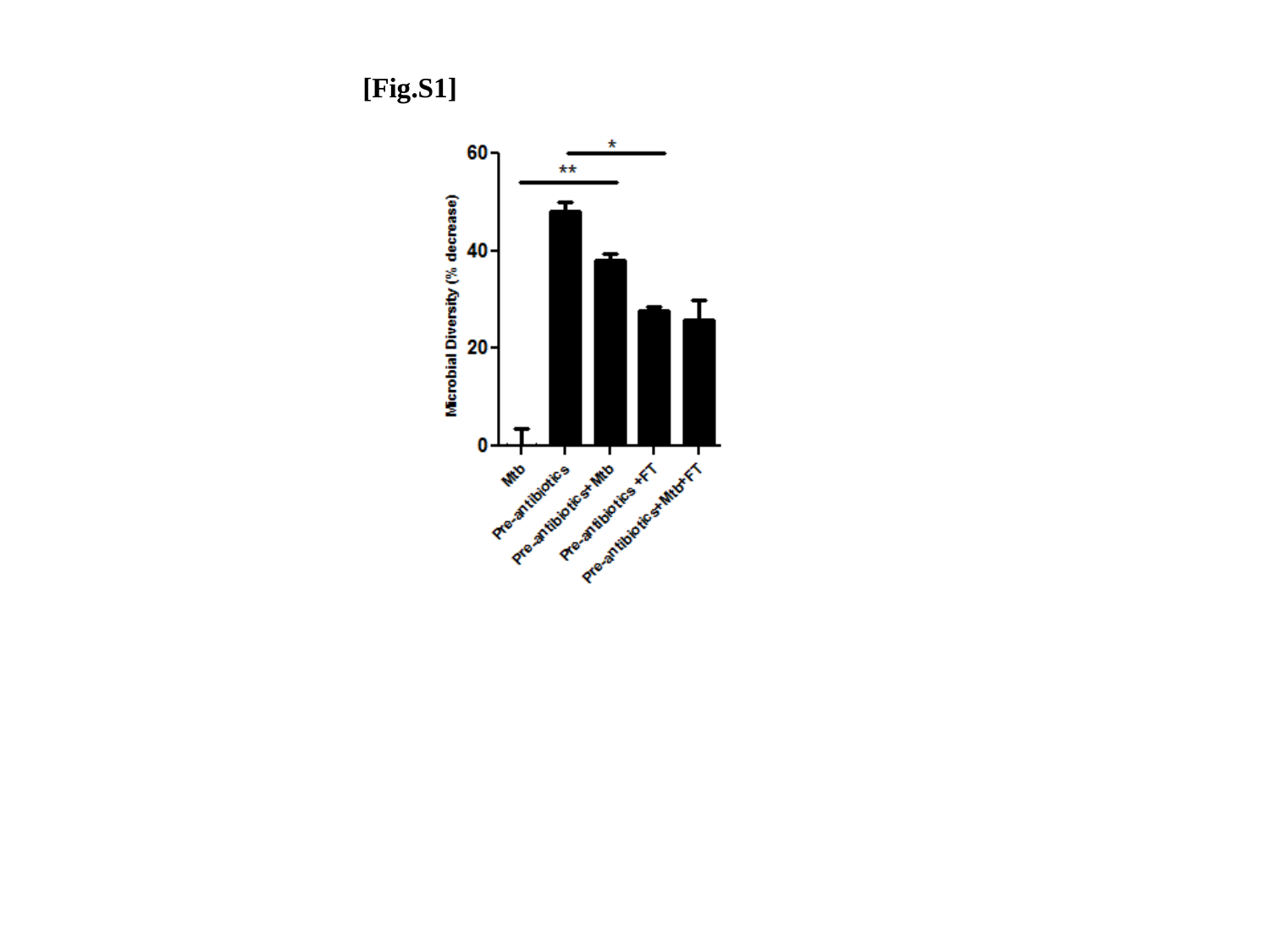

[Fig.S1]

## Slide 4
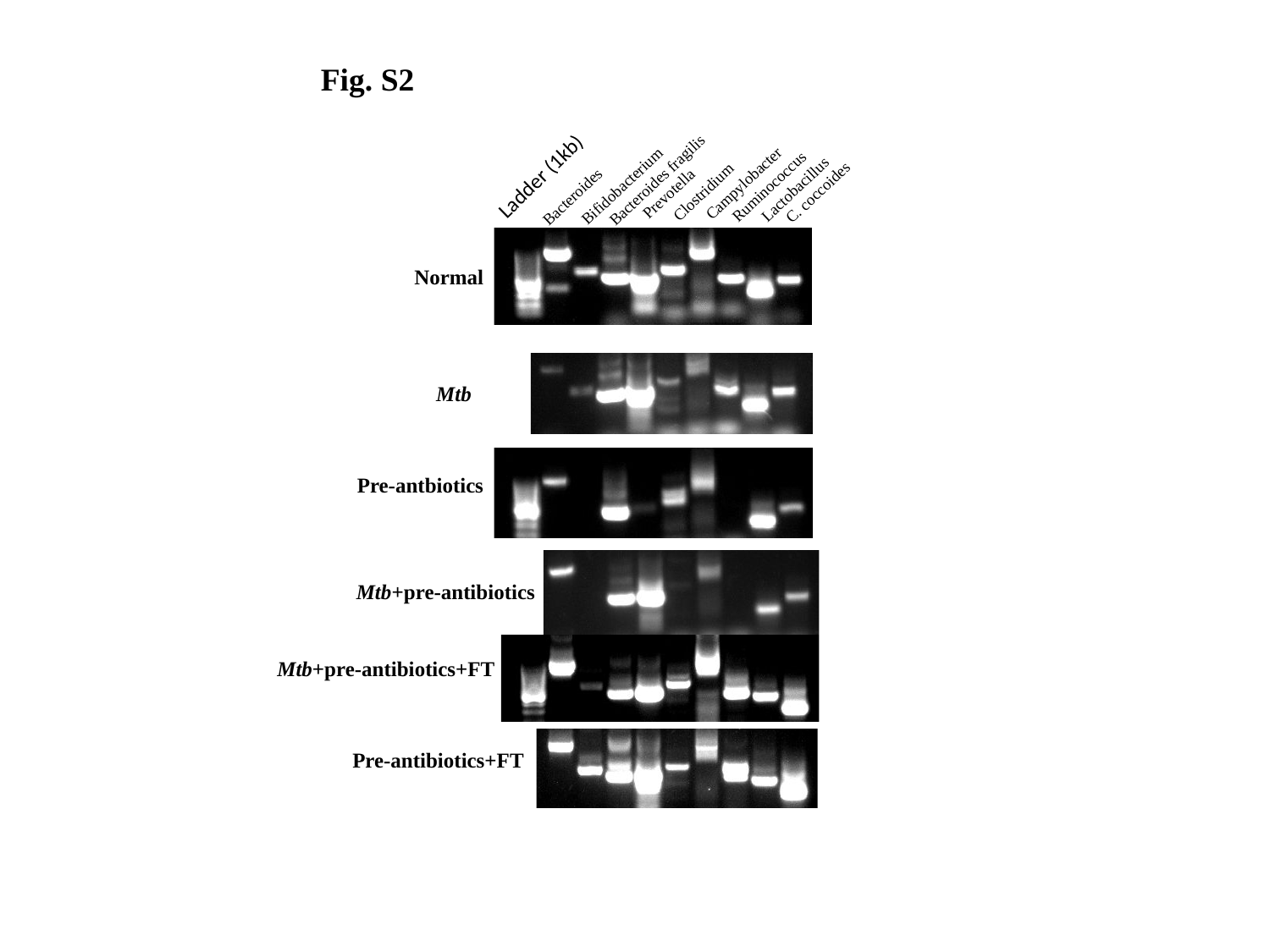

Fig. S2
Ladder (1kb)
Bacteroides fragilis
Campylobacter
Bifidobacterium
Ruminococcus
Lactobacillus
Clostridium
C. coccoides
Prevotella
Bacteroides
Normal
Mtb
Pre-antbiotics
Mtb+pre-antibiotics
 Mtb+pre-antibiotics+FT
Pre-antibiotics+FT

## Slide 5
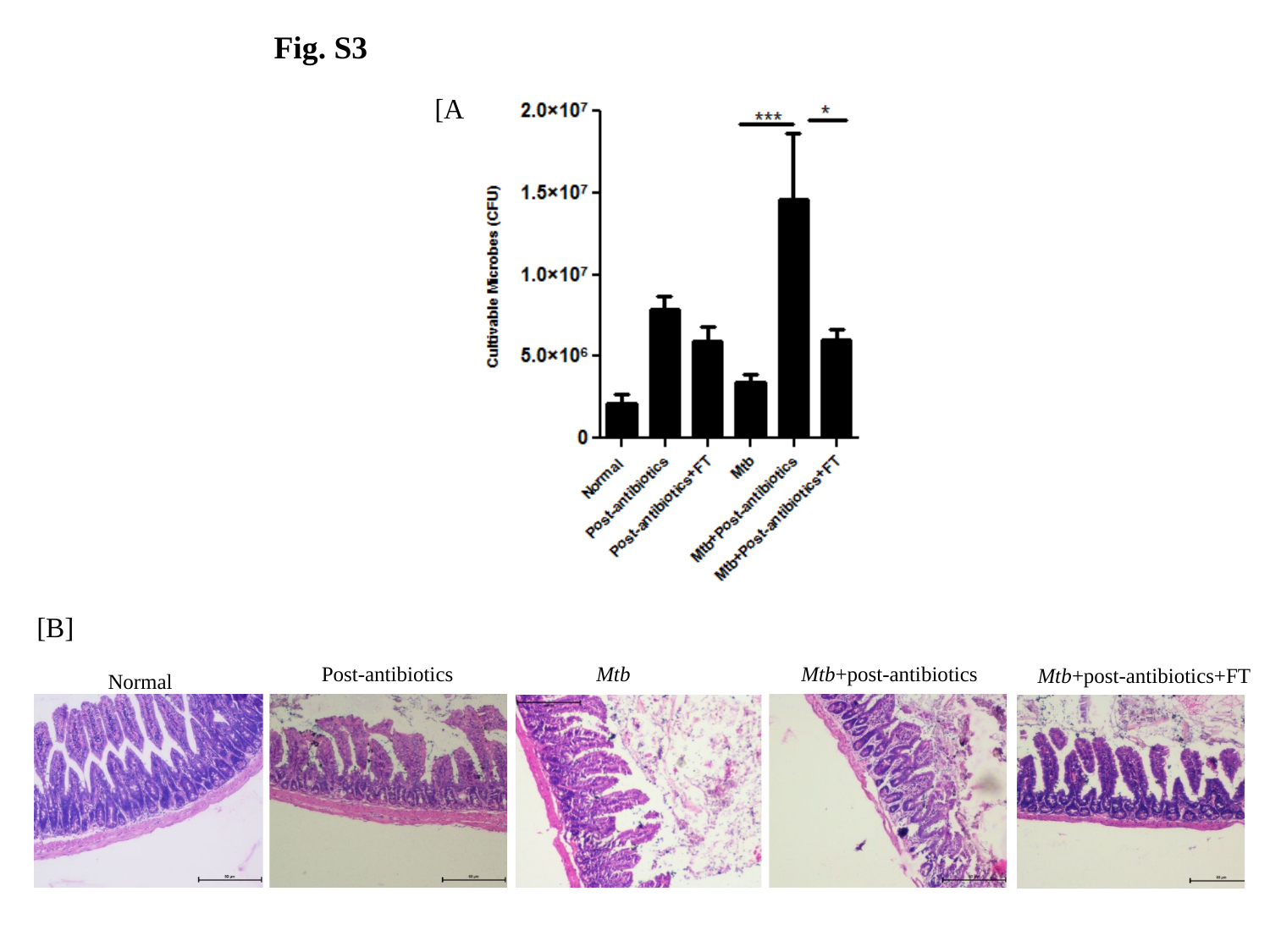

Fig. S3
[A]
[B]
Post-antibiotics
Mtb
Mtb+post-antibiotics
Mtb+post-antibiotics+FT
Normal
