# Supplementary material for: Alteration in the Gut Microbiota Provokes Susceptibility to Tuberculosis
Source: Front Immunol. 2016 Nov 28;7:529. doi: 10.3389/fimmu.2016.00529 (PMC5124573; doi:10.3389/fimmu.2016.00529)
Supplement: Supplementary file 2 [file Presentation_2.PDF]

Supplementary Information

Materials and methods

Mycobacterial strain and antigens. Mtb strain H37Rv was provided by Dr. VM Katoch, National JALMA Institute for Leprosy and Other Mycobacterial Diseases, Agra, India. *Mtb* was cultured in Middlebrook 7H9 broth containing glycerol (0.2%) and Tween-80 (0.05%), supplemented with albumin, dextrose and catalase. The viability of the bacteria was determined by plating on Middlebrook 7H11 medium, supplemented with oleic acid, albumin, dextrose and catalase and later counting the number of colony forming units (CFUs).

Abs and reagents. All standard chemicals, reagents, antibiotics used in the study were purchased from Sigma (St. Louis, MO) and Abs from BD Biosciences (San Diego, CA), unless and otherwise mentioned.

Sample collection. Fresh stool pellets were collected before mice were euthanized. Immediately, the contents of the cecum and from the distal 3cm of the small intestine (ileum), excluding the last 1cm proximal to the cecum, were also recovered by manual extrusion.

Quantification of gut microbes by RT-qPCR. DNA was isolated from fecal samples using ZR Fecal DNA Miroprep kit, according to manufacturer's instructions (Epigenetics, Irvin). DNA was quantified with the help of NanoDrop spectrophotometer. A260/A280 ratio of all samples was in the range of 1.90 to 2.00. The final reaction of RT-qPCR was performed in a volume of 10 μl, consisting of 1x SYBR green, 0.2 μM forward primer, 0.2 μM reverse primer and 50-100 ng of cDNA. Reactions were performed at 95ºC for 15s and 60ºC for 1 min for 40 cycles in Applied Biosystems step one PCR (Waltham, MA). PCR program was set according to the manufacture instructions. Analysis was done by comparative Ct method, whereas Ct values were normalized against universal control. Expression unit were expressed as $2^{-(\Delta CT*10000).}$ Primers

were used for detection of <u>*Bacteroides, Bifidobacterium, Campylobacter, Lactobacillus, Enterococcus.*</u>

Primer	Sequence	Reference
Bacteroides FW<br>*Bacteroides* RW	GAGAGGAAGGTCCCCCAC<br>CGCTACTTGGCTGGTTCAG	(Layton et al., 2006)
Bifidobacterium FW<br>*Bifidobacterium* RW	CGGGTGAGTAATGCGTGACC<br>TGATAGGACGCGACCCCA	(Tuomisto et al., 2013)
Campylobacter FW<br>*Campylobacter* RW	GGATGACACTTTTCGGAG<br>AATTCCATCTGCCTCTCC	(Rinttila et al., 2004)
Lactobacillus FW<br>*Lactobacillus* RW	AGCAGTAGGGAATCTTCCA<br>CACCGCTACACATGGAG	(Rinttila et al., 2004)
Enterococcus FW<br>*Enterococcus* RW	CCCTTATTGTTAGTTGCCATCATT<br>ACTCGTTGTACTTCCCATTGT	(Rinttila et al., 2004)

Identification of gut microbes by PCR. DNA was isolated from fecal samples using ZR Fecal DNA Miroprep kit, according to manufacturer's instructions (Epigenetics, USA). DNA was quantified with the help of NanoDrop spectrophotometer. A260/A280 ratio of all samples was in the range of 1.90 to 2.00. The final reaction of PCR was performed in a volume of 20 µl, consisting of 5X firepol PCR mastermix, 0.2 µM forward primer, 0.2 µM reverse primer and 50 ng gDNA. Reactions were performed at 95ºC for 45s, 55ºC for 45s and 72ºC for 30s for 30 cycles using Eppendorf gradient PCR. PCR program was set according to the manufacture's instructions (Solis Biodyne, Estonia). Primer sequences are mentioned below (Rekha et al., 2006, Matsuki et al., 2002)

<u>Primers Sequence:</u>

Primer	Sequence	Reference
Bacteroides FW<br>*Bacteroides* RW	GGGGTTCTGAGAGGAAG<br>ACCCCCCATTGTACCAC	(Rekha et al., 2006)
*Bifid*obacterium FW<br>*Bifidobacterium* RW	CTCCTGGAAACGGGTGG<br>GGTGTTCTTCCCGATATCTACA	(Matsuki et al., 2002)
Bacteroides fragilis FW<br>*Bacteroides fragilis* RW	ATAGCCTTTCGAAAGRAAGAT<br>CCAGTATCAACTGCAATTTTA	(Matsuki et al., 2002)
Prevotella FW<br>*Prevotella* RW	CACRGTAAACGATGGATGCC<br>GGTCGGGTTGCAGACC	(Matsuki et al., 2002)
C. coccoides FW<br>*C. coccoides* RW	AAATGACGGTACCTGACTAA<br>CTTTGAGTTTCATTCTTGCGAA	(Matsuki et al., 2002)
Lactobacillus FW<br>*Lactobacillus* RW	TGCCTAATACATGCAAGTCGA<br>GTTTGGGCCGTGTCTCAGT	(Rekha et al., 2006)
Ruminococcus FW<br>*Ruminococcus* RW	CCTCTGACCGCTCTTTAATCGGAGCTTTCCTTC<br>CCAGTTATCGGTCCCACCTTCGGCAGCT	(Rekha et al., 2006)
Clostridium FW<br>*Clostridium* RW	CTCAACTTGGGTGCTGCATTT<br>ATTGTAGTACGTGTGTAGCCC	(Rekha et al., 2006)
Campylobacter FW<br>*Campylobacter* RW	AGGGAATATTGCGCAATGGGGGAAA<br>TTACTAGCGATTCCGGCTTCATGC	(Rekha et al., 2006)

Histopathology. Cecal tips or spleen and lung sections were fixed in 10% neutral buffered formalin overnight. Fixed tissues were embedded in paraffin and cut into sections. Tissues were stained with hematoxylin and eosin (H&E). Granulomatous region in lungs was calculated by microscopy with the help of eye piece graticule.

Supplementary Figures

Fig. S1. Faecal transplantation partially reconstituted the gut microbiota. Pre-antibiotics mice were administered 5 doses of FT, 15d prior to sacrificing animals. Later, microbial diversity was

assessed in the fecal samples. Data shown as mean±SEM are representative of 2 independent experiments (n=4-5 animals/group). **p<0.01.

Fig. S2. Antibiotics treatment altered the gut microbiota. Mice treated with pre-antibiotics were infected with Mtb. After 42d, fecal sample were collected to isolate DNA. Later, microbes were identified by PCR.

Fig. S3. Faecal transplantation reconstituted the gut microbiota. (A) Post-antibiotics mice were administered 5 doses of FT, 15d prior to sacrificing animals. Later, (A) enumeration of cultivable microbes was assessed in the fecal samples. (B) Histopathology sections of ileum were H and E stained and photomicrographs are shown at 100X magnification. Data shown as mean±SEM are representative of 2 independent experiments (n=4-5 animals/group). *p<0.05, ***p<0.001.

References

Layton, A., Mckay, L., Williams, D., Garrett, V., Gentry, R., and Sayler, G. (2006). Development of Bacteroides 16S rRNA gene TaqMan-based real-time PCR assays for estimation of total, human, and bovine fecal pollution in water. *Appl Environ Microbiol* 72**,** 4214-4224.

Matsuki, T., Watanabe, K., Fujimoto, J., Miyamoto, Y., Takada, T., Matsumoto, K., Oyaizu, H., and Tanaka, R. (2002). Development of 16S rRNA-gene-targeted group-specific primers for the detection and identification of predominant bacteria in human feces. *Appl Environ Microbiol* 68**,** 5445-5451.

Rekha, R., Rizvi, M.A., and Jaishree, P. (2006). Designing and validation of genus-specific primers for human gut flora study. *Electron J Biotechnol* 9, 505–511.

Rinttila, T., Kassinen, A., Malinen, E., Krogius, L., and Palva, A. (2004). Development of an extensive set of 16S rDNA-targeted primers for quantification of pathogenic and indigenous bacteria in faecal samples by real-time PCR. *J Appl Microbiol* 97**,** 1166-1177.

Tuomisto, S., Karhunen, P.J., and Pessi, T. (2013). Time-dependent post mortem changes in the composition of intestinal bacteria using real-time quantitative PCR. *Gut Pathog* 5**,** 35.
